# Recent Advances in the Ecology of Bloom-Forming *Raphidiopsis* (*Cylindrospermopsis*) *raciborskii*: Expansion in China, Intraspecific Heterogeneity and Critical Factors for Invasion

**DOI:** 10.3390/ijerph20031984

**Published:** 2023-01-21

**Authors:** Ling Zheng, Yang Liu, Renhui Li, Yiming Yang, Yongguang Jiang

**Affiliations:** 1 Department of Biological Sciences and Technology, School of Environmental Studies, China University of Geosciences, Wuhan 430074, China; 2 College of Life Sciences, Henan Normal University, Xinxiang 453007, China; 3 College of Life and Environmental Sciences, Wenzhou University, Wenzhou 325035, China; 4 Guangdong Engineering Research Center of Oral Restoration and Reconstruction, Guangzhou Key Laboratory of Basic and Applied Research of Oral Regenerative Medicine, Affiliated Stomatology Hospital of Guangzhou Medical University, Guangzhou 510182, China

**Keywords:** cyanobacterial blooms, climate warming, cylindrospermopsin, microbial invasion, microevolution, nutrient fluctuations

## Abstract

Water blooms caused by the invasive cyanobacterium *Raphidiopsis raciborskii* occur in many reservoirs in the tropical and subtropical regions of China. In recent decades, this species has spread rapidly to temperate regions. Phenotypic plasticity and climate warming are thought to promote the worldwide dispersion of *R. raciborskii*. However, investigations into the genetic and phenotypic diversities of this species have revealed significant intraspecific heterogeneity. In particular, competition between *R. raciborskii* and *Microcystis aeruginosa* was highly strain dependent. Although the concept of an ecotype was proposed to explain the heterogeneity of *R. raciborskii* strains with different geographic origins, microevolution is more reasonable for understanding the coexistence of different phenotypes and genotypes in the same environment. It has been suggested that intraspecific heterogeneity derived from microevolution is a strong driving force for the expansion of *R. raciborskii*. Additionally, temperature, nutrient fluctuations, and grazer disturbance are critical environmental factors that affect the population establishment of *R. raciborskii* in new environments. The present review provides new insights into the ecological mechanisms underlying the invasion of *R. raciborskii* in Chinese freshwater ecosystems.

## 1. Introduction

**Figure 1:**
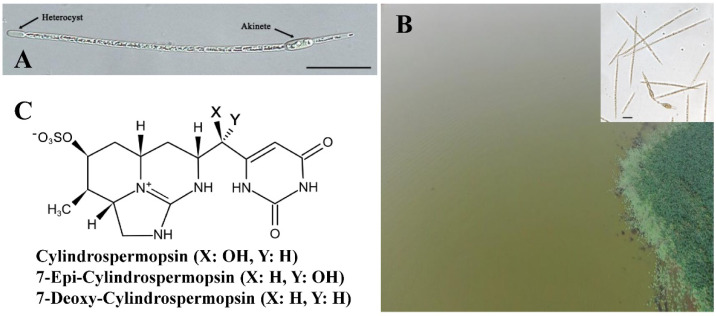
Cell differentiation of *R. raciborskii* filament (**A**), aerial view of the *R. raciborskii* bloom (**B**), and chemical structure of cylindrospermopsins (**C**). Inset in (**B**) is a microscopic graph of *R. raciborskii* bloom. Scale bars in (**A**,**B**), 10 µm.

Cyanobacterial blooms are severe environmental problems in eutrophic freshwater ecosystems [1,2]. *Raphidiopsis raciborskii* (previously known as *Cylindrospermopsis raciborskii*) is a filamentous bloom-forming cyanobacterium belonging to Nostocales with heterocysts and akinetes (Figure 1A). Upon the occurrence of an *R. raciborskii* bloom, a significant amount of cell filaments usually distributes evenly in the water column (Figure 1B). As a major producer of hepatotoxic cylindrospermopsin (CYN) and its analogs (Figure 1C) [3,4,5,6], *R. raciborskii* often proliferates in lakes or reservoirs in tropical and subtropical zones [7,8], posing a significant threat to ecological safety. In the past decades, the occurrence frequency of this species has been significantly increased over the world, including both subtropical and temperate zones of the globe [7,9,10]. Therefore, *R. raciborskii* was suggested to be an invasive cyanobacterium, and much attention has been paid to its dispersion routes and adaptation mechanisms [9,11,12].

Based on the phylogenetic relationship of strains isolated worldwide, three hypotheses have been proposed for the biogeographic origin and global dispersion of *R. raciborskii* [7]. First, *R. raciborskii* was suggested to be derived from tropical regions in Africa due to its temperature dependence and the high diversity of African strains [13]. After invasion and colonization in Australia, *R. raciborskii* was dispersed throughout Eurasia and America [13]. Similarly, a recent study on the biogeography of *R. raciborskii* supports central Africa as the original dispersion center for nontoxic strains of this species [14]. The ability to produce CYN and paralytic shellfish poison (PSP) is thought to be acquired after dispersion to new settlements in tropical Africa and the South American continent [14]. Second, it was thought that *R. raciborskii* was extinct in most areas of the world during the Pleistocene Ice Age, except for the warm refuges of each continent at low latitudes [15]. The surviving *R. raciborskii* strains were dispersed to high-latitude areas when the global climate became warm. Third, *R. raciborskii* was thought to be derived from a tropical region in America and dispersed to other continents [11]. Considering that phylogeographic analysis was strongly affected by the genetic variation of the strains used in different studies [16], sufficient strains from different continents and environments should be investigated to clarify the dispersion route of *R. raciborskii*.

The invasion success of *R. raciborskii* was mainly ascribed to its phenotypic plasticity and growth optimization under different environments [9,17,18,19,20]. *R. raciborskii* is tolerant to low and high light intensity and a wide range of temperatures [19,21,22,23,24,25]. This species has flexible strategies for utilizing various forms of nitrogen (N), such as accumulating nitrogen as cyanophycin [26] and switching to N_2_ fixation under N depletion [27,28,29]. With a strong affinity for phosphate, *R. raciborskii* is capable of absorbing and storing a lot of phosphorus (P) [18,30]. After depletion of inorganic P, dissolved organic phosphorus can be used by *R. raciborskii* through the secretion of alkaline phosphatase (ALP) [31,32]. Thus, *R. raciborskii* can be dominant in the phytoplankton community at both very low and high N:P ratios [33]. In addition, it has been suggested that this species has a competitive advantage over other bloom-forming cyanobacteria under N- or P-limited conditions [30,34,35]. For example, toxic *R. raciborskii* is favored by the allelopathic effects of CYN on the surrounding phytoplankton [36,37,38,39].

Recent investigations have revealed high genetic and phenotypic diversity within the population of *R. raciborskii* [40,41,42]. The adaptation mechanisms of different strains were divergent and the competitive advantage of *R. raciborskii* against *Microcystis aeruginosa* was strain dependent [43,44,45,46]. These findings indicated apparent intraspecific heterogeneity in *R. raciborskii*. A comprehensive investigation of *R. raciborskii* diversity and dispersion in local ecosystems can provide new insights into its invasion success. *R. raciborskii* has been detected in many lakes and reservoirs in China [8,35,47,48,49,50,51,52]. The recent expansion of *R. raciborskii* in Chinese freshwater bodies, its intraspecific heterogeneity, and critical factors for its invasion are the focus of this review.

## 2. Distribution and Dispersion in China

**Figure 2:**
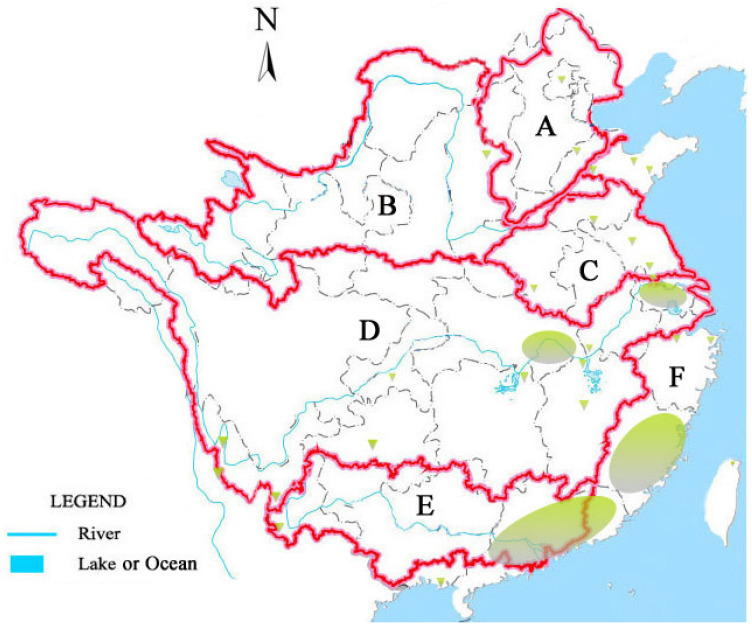
Distribution of *R. raciborskii* in major river basins of China. A, Haihe River basin; B, Huanghe River basin; C, Huaihe River basin; D, Yangtze River basin; E, Zhujiang River basin; F, basins of southeastern rivers. Blue-green color, areas with presence of *R. raciborskii*.

The first reported *R. raciborskii* bloom in Chinese water bodies occurred in 2002 at 21°59′ N–22°25′ N and 113°05′ E–113°25′ E [53]. To date, the presence of this species has been observed from 20°09′ N to 39°59′ N and between 100°03′ E and 121°41′ E, that is, from the tropical to temperate regions of China (Table S1, Figure 2). *R. raciborskii* was distributed in the basins of Zhujiang River, Yangtze River, Huaihe River, Yellow River, Haihe River, Yongjiang River, Qiantang River, and in the southeastern rivers in Fujian and Taiwan (Figure 2). Dominance or high biomass of this species was observed in most of the basins, except in the Haihe and Qiantang River basins. The Haihe River basin is the northernmost line with the presence of *R. raciborskii* in China [50]; this species may have dispersed to this basin recently and has not been fully adapted to the local environment. Given that the Qiantang River basin is located in a subtropical zone with favorable temperature conditions for the proliferation of *R. raciborskii*, its distribution in this basin needs further investigation. Although historical data on *R. raciborskii* distribution are lacking, it should be noted that bloom events of this species have increased in recent decades. In 2006, only a low concentration of *R. raciborskii* filaments was observed in Liangzi lake (30°10′ N, 114°36′ N) [48]. However, its biomass reached 52.03 mg L^−1^, with an obvious water bloom between 2013 and 2017 [35]. This finding indicates that *R. raciborskii* has evolved from an occasional species to a dominant species in Liangzi lake, which is likely a typical succession process after the invasion of new water bodies.

**Figure 3:**
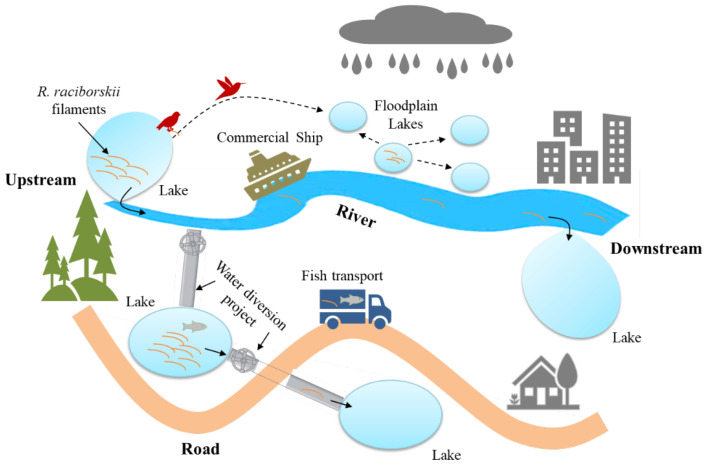
Probable dispersion routes for *R. raciborskii*.

The wide distribution of *R. raciborskii* in the same basin (Figure 2) indicated that this species might be dispersed from upstream lakes to downstream lakes through the river (Figure 3). On the other hand, lakes located separately in floodplain could be linked by large floods, creating opportunities for *R. raciborskii* to invade new environments. Water diversion projects (WDPs) also provide an important dispersion route for aquatic organisms. For example, fish invasion has occurred through the south-to-north WDP in China [54]. This WDP is designed to divert water from humid subtropical areas to arid or semi-arid temperate areas and thus is favorable for the dispersion of *R. raciborskii*. Five lakes in the Huaihe River basin were linked with three reservoirs and one pond in the Yellow and Haihe River basins by the east route of the south-to-north WDP (Table S1), and abundant *R. raciborskii* were observed in all of these water bodies. In addition, the filaments of this species can be conveyed between lakes via natural water used for the transport of aquatic products and ballast water of commercial ships [7]. Owing to their high survivability, the akinetes of *R. raciborskii* are likely to be transported by migratory birds via feet or gut carryover [55].

CYN is synthesized by a series of enzymes encoded by *cyr* genes [56]. Characterizing the distribution and variation of *cyr* genes can provide insights into the dispersion of CYN-producing *R. raciborskii* strains. A systematic investigation into the sequences of *cyrI* and *cyrJ* genes in Chinese water bodies divided them into four and three main sequence types, respectively (Table 1) [48]. It was shown that low-latitude reservoirs contain more sequence types. Given that Itype4a^f^ and Itype4a^r^ are Itype1 variants with transposition elements, and that Jtype2b is a Jtype2a variant with a deletion of six nucleotides, the Tiegang reservoir presumably contains all the ancestral *cyrI* and *cyrJ* sequence types. This finding indicated that low-latitude lakes in Southeast China are potential dispersion centers of toxic *R. raciborskii*. Similarly, the tropical regions in Southeast China were also thought to be a glacial refuge for higher plant species [57]. These findings provide further evidence for the refuge hypothesis about *R. raciborskii* dispersion [15]. PSP-producing *R. raciborskii* strains were also isolated and identified from Chinese water bodies [58,59]. Phylogenetic analysis revealed that these strains were closely related to strains from the North and South American continents [58]. This finding may imply the dispersion of PSP-producing strains from America to China because the ability of *R. raciborskii* to produce PSP was proposed to originate from America [14].

## 3. Intraspecific Heterogeneity

### 3.1. Phenotypic and Genetic Diversity

In previous studies on the environmental adaptability of *R. raciborskii*, it has often been regarded as a homogenous population because of the highly similar 16S rRNA genes of different strains. For example, most present studies suggest that *R. raciborskii* is a mesophile with a tolerance to low temperatures [7,19,60,61]. However, laboratory experimentation has found that several strains maintain a high growth rate at 15 °C, while the growth of some strains is completely inhibited under low-temperature conditions [17,47]. These findings indicate that low-temperature tolerance is not an intrinsic characteristic of *R. raciborskii* and that significant intraspecific heterogeneity exists for the temperature adaptability of this species. Similarly, the intraspecific heterogeneity of *R. raciborskii* has also been found in its response to light intensity [9,62,63] and conductivity [64], as well as its strategies for the utilization of N [26,27] and P [41]. In addition, varied growth rates, morphologies, and toxicities have been observed in genetically similar isolates of *R. raciborskii* [65,66].

In contrast to the 16S rRNA gene, the RNA polymerase C1 (*rpoC1*) gene and ITS-L sequence, which is the larger fragment of the inter-transcribed sequence of rRNA, are useful molecular markers for discriminating different *R. raciborskii* strains [58,67]. The strains were classified into nontoxic, CYN-producing, and PSP-producing clusters based on the phylogenetic analysis of *rpoC1* and ITS-L [58]. This result is supported by the genomic variations between *R. raciborskii* strains [16,42,68]. The relationship between genetic diversity and phenotypic variation remains to be clarified and requires further systematic investigation. 

The presence of *cyr* genes is the main genetic difference between CYN-producing and non-CYN-producing strains [48]. However, genomic variations were also found in genes associated with stress and adaptation, which are probably related to the physiological role of CYN [42]. In fact, CYN has been shown to contribute to the successful invasion and survival of *R. raciborskii* [4,5]. For example, CYN-producing strains have a competitive advantage under nutrient-replete conditions [69]. In comparison to nontoxic *R. raciborskii*, toxic strains have a more efficient response to inorganic phosphorus and could become dominant in the community [70]. A recent study also found that toxic strains were more competitive under Fe-starved conditions [71].

Previous investigations have revealed that the cell quotas of CYN varied significantly between *R. raciborskii* strains [65,66,72]. However, the genetic basis of this finding remains unknown. As aforementioned, sequence variations were observed for *cyrI* and *cyrJ* genes, which encodes a hydroxylase and a sulfotransferase, respectively [48,73,74,75]. These two enzymes catalyze tailoring reactions in the last two steps of CYN biosynthesis [56]. An inactivated mutation of *cyrI* inhibits the synthesis of CYN, leading to an accumulation in the intermediate product 7-deoxy-CYN [76]. Therefore, variations in *cyr* genes may change the activity of the enzymes encoded by them and further affect CYN synthesis efficiency.

### 3.2. Competition between R. raciborskii and M. aeruginosa

*M. aeruginosa* is the dominant species in most eutrophic water bodies, both in China and worldwide [51,77]. Competition against *M. aeruginosa* is inevitable for *R. raciborskii* during invasion. However, recent culture experiments have produced inconsistent results regarding the competition between *R. raciborskii* and *M. aeruginosa* (Table 2). Under light- or P-limited conditions, both *R. raciborskii* and *M. aeruginosa* are likely to dominate the mixed culture of these two species with equal starting biovolumes [45]. *R. raciborskii*, with N-fixation capability, was a more successful invader than *M. aeruginosa* when N was depleted in batch culture [35]. Likewise, the model prediction of species competition outcomes is strongly affected by the growth variability of strains [78]. These findings demonstrate the intraspecific heterogeneity for *R. raciborskii* and *M. aeruginosa* [44].

### 3.3. Ecotype and Microevolution

In several studies, *R. raciborskii* was assumed to have evolved into different ecotypes with special physiological characteristics in its original habitats, and the geographic dispersion of this species is a dynamic selection process for existing ecotypes [60,61,72]. The concept of an ecotype is reasonable for understanding the intraspecific heterogeneity of *R. raciborskii* in different environments but not for explaining the coexistence of different phenotypes and genotypes in the same environment [16,65]. Heterogeneity between coexisting strains is better defined as microevolution, the concept of which refers to minor variations within species [80]. Microevolution is the source of adaptive variation for organisms, and the accumulation of microevolution may further lead to new speciation. The ecotypes of *R. raciborskii* were presumably established by the natural selection of heterogeneous strains generated during microevolution.

## 4. Critical Factors for Invasion

### 4.1. Temperature and Effective Accumulated Temperature (EAT)

Temperature is an important ecological factor that drives the global distribution of *R. raciborskii* [10,13,81]. Climate warming is expected to increase the biomass of this species and promote its dispersion in new habitats (Table 3). In addition to its proliferation in warm areas, the dominance and bloom of this species were observed in cool water between 10 °C and 15 °C [22,47]. This finding indicates that *R. raciborskii* is likely to disperse and survive in most freshwater bodies in China. Under nutrient-replete conditions, the potential biomass that *R. raciborskii* can reach is probably determined by the Effective Accumulated Temperature (EAT) affecting the heat that organisms can obtain during multiplication seasons. To date, there are no available data on the effects of EAT on the growth of *R. raciborskii*. In China, the EAT varies with latitude from south to north and altitude from east to west. It is necessary to investigate the relationship between EAT and the distribution of *R. raciborskii* to assess the bloom risk this species presents. 

### 4.2. Nutrient Fluctuations

The coexistence of multiple species is frequently observed during cyanobacterial blooms in eutrophic water bodies [51]. This finding implies that competitive exclusion does not occur, which may be attributed to nutrient repletion in the environment. For example, *R. raciborskii* may invade into an *M. aeruginosa*-dominated culture and survive with relatively low biomass [35]. The coexistence of *R. raciborskii* and *M. aeruginosa* has been found in many subtropical water bodies located in the Yangtze River basin [35]. Sometimes, *R. raciborskii* becomes the dominant species in the phytoplankton community. Considering intraspecific heterogeneity, it cannot be predicted that *R. raciborskii* will eventually compete against *M. aeruginosa*.

Niche and fitness differences are thought to determine invasion success in bacterial communities [82]. Field investigations have demonstrated that *Raphidiopsis*, rather than *Microcystis*, benefit from low P concentrations [83]. This finding was supported by laboratory experiments indicating that *R. raciborskii* could be advantageous under P-limited conditions [34,84]. This advantage may be enhanced using the allelopathic effects of CYN, which can induce extracellular ALP production in other phytoplankton species [39,85]. In addition, stoichiometric analysis revealed a lower metabolic cost of synthesizing CYN than that of producing ALP [39]. Moreover, N limitation may favor the growth of *R. raciborskii* strains with N-fixation capability. However, previous studies have claimed that this high-energy-consuming function cannot support the proliferation of *R. raciborskii* [40]. Nutrient fluctuations frequently occur in natural water bodies [84,86,87]. Intermittent P and N limitations may create opportunities for *R. raciborskii* to become dominant in the phytoplankton community [87] and proliferate when nutrients are repleted (Table 3).

### 4.3. Grazer Disturbance

Local grazers have a significant impact on the structure of phytoplankton communities [88]. *Daphnia* feeding on *R. raciborskii* can hamper the population establishment of this species [88,89], whereas copepods, preferentially grazing on non-*Raphidiopsis* species, can promote the persistence of *R. raciborskii* [90,91] (Table 3). Therefore, a low grazing pressure or loss of top-down control is a critical factor for the invasion success of *R. raciborskii* [88]. In addition, CYN-producing strains may be more advantageous because of the toxicity of CYN on zooplankton [5].

## 5. Conclusions

*R. raciborskii* has spread widely in Chinese freshwater bodies in recent decades. In addition to its adaptability to various environments, the intraspecific heterogeneity of *R. raciborskii* presumably has a significant contribution to the species’ rapid expansion. Temperature, nutrient fluctuations, and grazer disturbance are important factors that affect the invasion success of *R. raciborskii*.

## 6. Perspectives

### 6.1. Hypothesis for the Invasion Success of R. raciborskii

Field populations of *R. raciborskii* are mixtures of heterogeneous strains. When the mixtures were dispersed in new environments, strains with strong adaptability could survive under natural selection and evolve into ecotypes with specific genetic and phenotypic characteristics. Some ecotypes of *R. raciborskii* are likely to proliferate and form blooms under appropriate conditions. In summary, the invasion success of *R. raciborskii* may be ascribed to sufficient intraspecific heterogeneity, rather than the adaptability of individual strains.

### 6.2. Future Outlook

Given that *R. raciborskii* has a growth advantage under nutrient-limited conditions, the expansion of this species may benefit from a reduction in P and N during the remediation of lake eutrophication [92]. To enhance our understanding of the bloom-forming mechanisms of *R. raciborskii*, knowledge of the intraspecific heterogeneity of *R. raciborskii* should be strengthened in the future. To accurately discriminate between strain differences, quantitative traits should be defined and investigated systematically. The growth rate, photosynthetic rate, electron transfer rate, affinity for inorganic carbon, pigment content, extracellular ALP activity, toxin content, and critical requirements for nutrients, temperature, and light intensity, are candidate quantitative traits for *R. raciborskii*. Moreover, highly variable genetic markers, such as ITS-L, can be used to discriminate strain differences at the molecular level. The consistency between molecular markers and quantitative traits should be carefully evaluated. Moreover, it is crucial to identify genes controlling quantitative traits to provide more reliable genetic evidence for the heterogeneity of *R. raciborskii*.

When a specific field population of *R. raciborskii* is investigated, many strains should be isolated to fully capture their variability. Considering the effects of in-culture evolution on laboratory experiments [93], strains should be maintained under conditions similar to their original habitats, and their quantitative traits should be characterized after isolation as soon as possible. To develop a worldwide database describing the intraspecific heterogeneity of *R. raciborskii*, unified and standard experimental methods and procedures are required. Furthermore, attention should be paid to the effects of EAT on the distribution and heterogeneity of *R. raciborskii* for assessing its bloom risk.

## Figures and Tables

**Table 1:** Types of *cyrI* and *cyrJ* sequences from Chinese water bodies.

Water Body	Latitude (N), Longitude (E)	*cyrI*	*cyrJ*
Liangzi lake, Ezhou, Hubei	114°31′ E, 30°15′ N	Itype2	Not detected
Qiaodun lake, Daye, Hubei	114°43′ E, 30°14′ N	Itype4a^f^	Jtype2a
Chidong lake, Qichun, Hubei	115°25′ E, 30°06′ N	Itype4a^f^, Itype4a^r^	Jtype2a
Xianghu lake, Hangzhou, Zhejiang	120°12′ E, 30°7′ N	Itype1	Jtype2a
Qiandao lake, Hangzhou, Zhejiang	118°57′ E, 29°33′ N	Itype2	Jtype2a, Jtype2b
Dongzhen reservoir, Putian, Fujian	118°57′ E, 25°29′ N	Itype3	Jtype1
Shiyan reservoir, Shenzhen	113°54′ E, 22°42′ N	Itype1	Jtype1, Jtype2b
Tiegang reservoir, Shenzhen	113°53′ E, 22°37′ N	Itype1, Itype2, Itype3	Jtype1, Jtype2a, Jtype3

**Table 2:** Results of competition experiments using *R. raciborskii* and *M. aeruginosa*.

Strain Pair and Culture Condition		Dominant Strain	Reference
*R. raciborskii* FACHB 1096 vs. *M. aeruginosa* strain 205			
P substrates	K_2_HPO_4_, β-glycerol phosphate, (2-aminoethyl)-phosphinic acid, P-free	*R. raciborskii* FACHB 1096	[34]
	Glyphosate	*M. aeruginosa* strain 205	
*R. raciborskii* CS vs. *M. aeruginosa* LEA			
P and light	P-limitation	*R. raciborskii* CS	[45]
	Light-limitation	*R. raciborskii* CS	
*R. raciborskii* CP vs. *M. aeruginosa* MIRF			
P and light	P-limitation	*M. aeruginosa* MIRF	[45]
	Light-limitation	*M. aeruginosa* MIRF	
*R. raciborskii* N8 vs. *M. aeruginosa* FACHB905			
Temperature	16 °C, 24 °C, 32 °C	*M. aeruginosa* FACHB905	[44]
*R. raciborskii* N8 vs. *M. aeruginosa* (FACHB469 and 915) with a biovolume ratio of 30:1			
Temperature	16 °C, 24 °C, 32 °C	*R. raciborskii* N8 maintained initial advantages at 16 °C and 32 °C	[44]
		*M. aeruginosa* strains at 24 °C	
*R. raciborskii* ITEP-A1 vs. *M. aeruginosa* NPLJ-4			
pH and inorganic carbon	Aeration	*R. raciborskii* ITEP-A1	[79]
	Bicarbonate	*M. aeruginosa* NPLJ-4	
*R. raciborskii* NW-R vs. *M. aeruginosa* NW-M			
Growth capability	Multiple inoculation ratios	*R. raciborskii* NW-R	[35]
Four *M. aeruginosa* vs. eight *R. raciborskii* strains			
Growth capability	Simulated using a deterministic model	No absolute winner	[78]

**Table 3:** Adaptation of *R. raciborskii* to different ecological scenarios.

Scenarios	Level	Response	Consequence
Climate warming	Higher temperature	Growth promotion	Biomass increase and dispersion to new habitats
P fluctuation	Low concentration	ALP or CYN secretion	Become dominant
	High concentration	High P absorption	Biomass increase
N fluctuation	Low concentration	N fixation	Become dominant
	High concentration	Heterocyst inhibition and N absorption	Biomass increase
Grazing pressure	Grazer feeding on *R. raciborskii*	Growth inhibition	Biomass decrease
	Grazer feeding on non-*R. raciborskii* phytoplankton	Growth promotion	Become dominant

## Data Availability

The data presented in this study are available on request from the corresponding author.

## Supplementary Materials

The following supporting information can be downloaded at: https://www.mdpi.com/article/10.3390/ijerph20031984/s1, Table S1: Distribution of *Raphidiopsis raciborskii* in Chinese freshwater bodies. References [8,35,47,48,50,52,53,69,83,85,92,94,95,96,97,98,99,100,101,102,103,104,105,106,107,108] are cited in the supplementary materials.
