# Supplementary material for: Recent Advances in the Ecology of Bloom-Forming Raphidiopsis (Cylindrospermopsis) raciborskii: Expansion in China, Intraspecific Heterogeneity and Critical Factors for Invasion
Source: Int J Environ Res Public Health. 2023 Jan 21;20(3):1984. doi: 10.3390/ijerph20031984 (PMC9915880; doi:10.3390/ijerph20031984)
Supplement: Supplementary file 1 [file ijerph-20-01984-s001.zip › ijerph-2087383-supplementary.pdf]

Table S1 Distribution of *Raphidiopsis raciborskii* in Chinese freshwater bodies

Geographic origin	Latitude (N), Longitude (E)	Date of investigation	Abundance description	Reference
**Haihe river basin**				
Datun reservoir, Dezhou, Shandong [a]	37°16′, 116°12′	2015 – 2017	Occurrence	[1]
Fish pond, Beijing National Stadium, Beijing	39°59′, 116°24′	Aug., 2009	Occurrence	[2]
Five lakes/ponds, Beijing	39°53′ – 39°59′, 116°18′ – 116°29′	Sept., 2015	Occurrence	[3]
**Yellow River Basin**				
Jihongtan fish pond, Qingdao, Shandong [a]	36°20′, 120°13′	Nov., 2013	Dominant	[2]
Donghu reservoir, Jinan, Shandong [a]	36°52′, 117°20′	2017	Dominant	[1]
Shuangwangcheng reservoir, Weifang, Shandong [a]	37°08′, 118°43′	2017	Dominant	[1]
Jinyang lake, Taiyuan, Shanxi	37°46′, 112°30′	Aug., 2010	Occurrence	[2]
**Huaihe river basin**				
Nanwan reservoir, Xinyang, Henan	32°04′, 113°58′	Sept., 2018	Occurrence	[4]
Shaobo lake, Yangzhou, Jiangsu [a]	32°39′, 119°24′	Apr., 2013	0.01 – 0.06 mg L^{-1}	[5]
Gaoyou lake, Gaoyou, Jiangsu [a]	32°53′, 119°21′	Apr., 2013	0.01 – 0.02 mg L^{-1}	[5]
Baima lake, Huaian, Jiangsu [a]	33°14′, 119°07′	Apr., 2013	0.01 – 0.24 mg L^{-1}	[5]
Luoma lake, Suqian, Jiangsu [a]	34°03′, 118°13′	Apr., 2013	0 – 1.49 mg L^{-1}	[5]
Weishan lake, Xuzhou, Jiangsu [a]	34°34′, 117°17′	Apr., 2013	9.34 – 36 mg L^{-1}	[5]
**Qiantang river basin**				
Qiandao lake, Hangzhou, Zhejiang	29°35′, 118°59′	2012	Occurrence	[2]
Xianghu lake, Hangzhou, Zhejiang	30°08′, 120°13′	2012	Occurrence	[2]

Xihu lake, Hangzhou, Zhejiang	30°15′, 120°09′	2012	Occurrence	[2]
**Yongjiang river basin**				
Dongqian lake, Ningbo, Zhejiang	29°44′ – 29°47′, 121°37′ – 21°41′	2011	Dominant	[6]
**Southeastern rivers**				
13 reservoirs, Fujian	23°33′ – 28°20′, 115°50′ – 120°40′	2009 – 2010	Occurrence or Dominant	[7]
Shidou reservoir, Xiamen, Fujian	24°42′, 118°00′	2016 – 2018	Dominant, < 5 mg L^{-1} – > 7 mg L^{-1}	[8]
Tingxi reservoir, Xiamen, Fujian	24°48′, 118°08′	2016 – 2018	< 0.04 mg L^{-1}	[8]
Dongzhen reservoir, Putian, Fujian	25°28′ – 25°30′, 118°54′ – 118°59′	2009 – 2011	Dominant	[9,10]
Pond, Taipei, Taiwan	25°02′, 121°37′	2009 – 2011	Dominant from summer to autumn	[11]
**Yangtze river basin**				
Yilong lake, Shiping, Yunnan	23°39′ – 23°42′, 102°30′ – 102°38′	2013 – 2014	Dominant	[12]
Fish pond, Kunming, Yunnan	24°58′, 102°38′	Oct., 2006	Occurrence	[2]
Erhai lake, Dali, Yunnan	25°36′ – 25°58′, 100°05′ – 100°17′	2018 – 2019	0.00 – 2.49 mg L^{-1}	[13]
Xihu lake, Dali, Yunnan	26°01′, 100°03′	2018 – 2019	0.01 – 42.44 mg L^{-1}	[13]
Chenghai lake, Lijiang, Yunnan	26°33′, 100°39′	Dec. 2012	Occurrence	This study
Hongfeng reservoir, Guiyang, Guizhou	26°32′, 106°24′	2018 – 2019	Dominant	[14]
Nanpeng reservoir, Chongqing	29°19′, 106°41′	Apr. 2018 – Sept. 2018	Dominant, 2.53×10^8 cells L^{-1}	[15]
Junshan lake, Nanchang, Jiangxi	28°32′, 116°18′	Sept., 2013	0.06 – 0.92 mg L^{-1}	[5]
Poyang lake, Nanchang, Jiangxi	28°32′, 116°18′	Sept., 2013	Occurrence	[5]
Zhuhu lake, Nanchang, Jiangxi	29°09′, 116°41′	Sept., 2013	0.01 mg L^{-1}	[5]
Xinmiao lake, Jiujiang, Jiangxi	29°21′, 116°09′	Sept., 2013	Occurrence	[5]
Qili lake, Jiujiang, Jiangxi	29°40′, 115°55′	Sept., 2013	Occurrence	[5]

Dongting lake, Yueyang, Hunan	29°17′, 112°36′	May, 2013	0.01 mg L^{-1}	[5]
Southern lake, Yueyang, Hunan	29°20′, 113°07′	May, 2013	0.03 mg L^{-1}	[5]
Lushui reservoir, Chibi, Hubei	29°41′, 113°55′	May, 2006; 2012	Occurrence	[2]
Honghu lake, Honghu, Hubei	29°55′, 113°25′	Jun., 2013	0.14 mg L^{-1}	[5]
Xiliang lake, Xianning, Hubei	29°58′, 114°04′	Jun., 2013	0.11 mg L^{-1}	[5]
Futou lake, Xianning, Hubei	30°00′, 114°16′	Jun., 2013	0.08 mg L^{-1}	[5]
Kuzhu lake, Wuhan, Hubei	30°06′, 114°12′	2013 – 2017	21.71 mg L^{-1}	[4]
Chidong lake, Qichun, Hubei	30°07′, 115°24′	2006.08	Occurrence	[2]
Liangzi lake, Ezhou, Hubei	30°10′, 114°36′	2006; Sept., 2011	Occurrence	[2]
		2013 – 2017	52.03 mg L^{-1}	[4]
Wan lake, Wuhan, Hubei	30°19′, 114°01′	2013 – 2017	3.92 mg L^{-1}	[4]
Niushan lake, Wuhan, Hubei	30°19′, 114°33′	Jun., 2013	0.12 – 0.91 mg L^{-1}	[5]
Sanshan lake, Ezhou, Hubei	30°19′, 114°46′	Jun., 2013	0.38 – 1.07 mg L^{-1}	[5]
Wangjia lake, Wuhan, Hubei	30°21′, 113°53′	2013 – 2017	2.69 mg L^{-1}	[4]
Guojia lake, Wuhan, Hubei	30°22′, 114°11′	2013 – 2017	0.14 mg L^{-1}	[4]
Guanlian lake, Wuhan, Hubei	30°23′, 114°02′	2013 – 2017	0.18 mg L^{-1}	[4]
Jindui lake, Wuhan, Hubei	30°23′, 113°57′	2013 – 2017	0.62 mg L^{-1}	[4]
Tangxun lake, Wuhan, Hubei	30°24′, 114°22′	2013 – 2017	0.8 mg L^{-1}	[4]
Wulang lake, Wuhan, Hubei	30°24′, 114°05′	2013 – 2017	0.17 mg L^{-1}	[4]
Zhulin lake, Wuhan, Hubei	30°26′, 114°09′	2013 – 2017	1.2 mg L^{-1}	[4]
Changhu lake, Jingzhou, Hubei	30°27′, 112°26′	Jun., 2013	0.38 mg L^{-1}	[5]
Wanjia lake, Wuhan, Hubei	30°28′, 114°10′	2013 – 2017	2.14 mg L^{-1}	[4]
Nanhu lake, Wuhan, Hubei	30°29′, 114°21′	Aug., 2006	Occurrence	[2]
Houguan lake, Wuhan, Hubei	30°31′, 114°00′	2013 – 2017	2.4 mg L^{-1}	[4]
Yanxi lake, Wuhan, Hubei	30°32′, 114°28′	2013 – 2017	1.1 mg L^{-1}	[4]

Donghu lake, Wuhan, Hubei	30°33′, 114°23′	Nov., 2006; 2012	Occurrence	[2]
Dongyin lake, Wuhan, Hubei	30°38′，114°12′	2013 – 2017	0.03 mg L^{-1}	[4]
Dongda lake, Wuhan, Hubei	30°39′, 114°10′	2013 – 2017	0.50 mg L^{-1}	[4]
Chaipo lake, Wuhan, Hubei	30°41′, 114°33′	2013 – 2017	0.02 mg L^{-1}	[4]
Mejiao lake, Wuhan, Hubei	30°43′, 114°08′	2013 – 2017	0.07 mg L^{-1}	[4]
Wu lake, Wuhan, Hubei	30°47′, 114°29′	2013 – 2017	0.08 mg L^{-1}	[4]
Huanghu lake, Susong, Anhui	30°01′, 116°19′	Jun., 2013	0.05 – 0.06 mg L^{-1}	[5]
Hengshan reservoir, Yixing Jiangsu	31°14′, 119°34′	2010 – 2011	Dominant in summer	[16]
Gucheng lake, Nanjing, Jiangsu	31°18′, 118°53′	Apr., 2013	6.24 – 8.99 mg L^{-1}	[5]
Yangcheng lake, Suzhou, Jiangsu	31°25′, 120°48′	2013 – 2017	0.01 mg L^{-1}	[4]
Shijiu lake, Nanjing, Jiangsu	31°28′, 118°52′	Apr., 2013	2.82 – 7.43 mg L^{-1}	[5]
Shijiu lake, Nanjing, Jiangsu	31°30′, 118°56′	2013 – 2017	4.47 mg L^{-1}	[4]
Wulihu, Wuxi, Jiangsu	31°32′, 120°14′	Oct., 2013	Occurrence	This study
Kuncheng lake, Changshu, Jiangsu	31°34′, 120°44′	Apr., 2013	0.01 mg L^{-1}	[5]
Changdang lake, Liyang, Jiangsu	31°37′, 119°33′	Apr., 2013	0.11 mg L^{-1}	[5]
Yueliangwan fish pond, Nanjing, Jiangsu	32°03′, 118°50′	Nov., 2007	Occurrence	This study
**Zhujiang river basin**				
Fish pond, Zhuhai, Guangdong	21°59′ – 22°25′, 113°05′ – 113°25′	2002 – 2003	Dominant	[17]
Hedi reservoir, Zhanjiang, Guangdong	21°46′, 110°19′	2003	Dominant in raining season	[18]
Fusha reservoir, Zhongshan, Guangdong	22°24′, 113°28′	Sept., 2006	Dominant	[2]
5 reservoirs, Shenzhen, Guangdong	22°24′ – 22°52′, 113°43′ – 114°38′	2006 – Jun., 2007	Occurrence or Dominant	[2]
Donghu, Guangzhou, Guangdong	23°14′, 113°14′	Sept., 2007	Occurrence	This study
20 reservoirs around Guangdong	20°09′ – 25°31′, 109°45′ – 117°20′	2010	0. 02% – 97. 07%, in total phytoplankton biomass	[19]

21 reservoirs, Dongguan, Guangdong	22°39′ – 23°09′, 113°31′ – 114°15′	2011 – 2012	0.1% – 90.3% in total phytoplankton biomass	[20]
Fish pond, Panyu, Guangdong	22°57′, 113°19′	May, 2012	Dominant	[2]
Zhenhai reservoir, Jiangmen, Guangdong	22°34′, 112°33′	Nov. 2014 – Oct. 2015	Dominant, 5.9 – 15.5 mg L^{-1}	[21]
Dashahe reservoir, Jiangmen, Guangdong	22°32′, 112°24′	2018	Dominant	[22]
Qiandeng lake, Foshan Guangdong	23°03′, 113°08′	2017 – 2018	Occurrence	[23]
115 reservoirs around Guangdong	20°09′ – 25°31′, 109°45′ – 117°20′	2016; 2018	$3.73 \times 10^6 – 1.83 \times 10^7$ cells L^{-1}	[24]
More than 40% of 120 reservoirs around Guangdong	20°09′ – 25°31′, 109°45′ – 117°20′	Summer, 2018	Occurrence or Dominant	[25]
Hongchaojiang, Beihai, Guangxi	21°49′, 109°09′	2018 – 2019	Dominant	[26]

Note: a, East Route of South-North Water Diversion Project.

References

1. Wang, Y. Variation of cyanobacteria community and in the main regulating reservoirs in Shandong province and the growth characteristics of *Cylindrospermopsis*. Master, Shandong University, Jinan, China, 2018 (in Chinese).
2. Jiang, Y.; Xiao, P.; Yu, G.; Shao, J.; Liu, D.; Azevedo, S.M.F.O.; Li, R. Sporadic distribution and distinctive variations of cylindrospermopsin genes in cyanobacterial strains and environmental samples from Chinese freshwater bodies. *Appl. Environ. Microbiol.* **2014**, *80*, 5219-5230, doi:10.1128/aem.00551-14.
3. Xie, J.; Yu, G.; Xu, X.; Li, S.; Li, R. The morphological and molecular detection for the presence of toxic *Cylindrospermopsis* (Nostocales, Cyanobacteria) in Beijing city, China. *J. Oceanol. Limnol.* **2018**, *36*, 263-272, doi:10.1007/s00343-018-6283-x.
4. Jia, N.; Yang, Y.; Yu, G.; Wang, Y.; Qiu, P.; Li, H.; Li, R. Interspecific competition reveals *Raphidiopsis raciborskii* as a more successful invader than *Microcystis aeruginosa*. *Harmful Algae* **2020**, *97*, 101858, doi:10.1016/j.hal.2020.101858.
5. Yang, Y.; Jiang, Y.; Li, X.; Li, H.; Chen, Y.; Xie, J.; Cai, F.; Li, R. Variations of growth and toxin yield in *Cylindrospermopsis raciborskii* under different phosphorus concentrations. *Toxins* **2016**, *9*, 13, doi:doi: 10.3390/toxins9010013.

6. Li, X.; Huo, S.; Zhang, J.; Xiao, Z.; Xi, B.; Li, R. Factors related to aggravated *Cylindrospermopsis* (cyanobacteria) bloom following sediment dredging in an eutrophic shallow lake. *Environ. Sci. Ecotechnol.* **2020**, *2*, 100014, doi:10.1016/j.ese.2020.100014.
7. Zheng, H. Risk assessment of cyanobacterial blooms in drinking water reservoirs of Fujian province. *Hua Xue Gong Cheng Yu Zhuang Bei* **2012**, 201-203 (in Chinese), doi:10.3969/j.issn.1003-0735.2012.06.067.
8. Gao, X.; Wang, W.; Ndayishimiye, J.C.; Govaert, L.; Chen, H.; Jeppesen, E.; Xue, Y.; Yu, X.; Yang, J. Invasive and toxic cyanobacteria regulate allochthonous resource use and community niche width of reservoir zooplankton. *Freshw. Biol.* **2022**, *67*, 1344-1356, doi:https://doi.org/10.1111/fwb.13921.
9. Zheng, H. Characteristics of phytoplankton community and nutrient conditions in Dongzhen reservoir. *Hua Xue Gong Cheng Yu Zhuang Bei* **2012**, 193-195+200 (in Chinese), doi:10.3969/j.issn.1003-0735.2012.05.062.
10. Jiang, Y.; Xiao, P.; Liu, Y.; Wang, J.; Li, R. Targeted deep sequencing reveals high diversity and variable dominance of bloom-forming cyanobacteria in eutrophic lakes. *Harmful Algae* **2017**, *64*, 42-50, doi:10.1016/j.hal.2017.03.006.
11. Yamamoto, Y.; Shiah, F.-K. Factors related to the dominance of *Cylindrospermopsis raciborskii* (cyanobacteria) in a shallow pond in northern Taiwan. *J. Phycol.* **2012**, *48*, 984-991, doi:10.1111/j.1529-

8817.2012.01184.x.

12. Wang, Z.F.; Zhang, W.; Yang, L.; Xu, Y.P.; Zhao, F.B.; Wang, L.Q. Characteristics of phytoplankton community and its relationship with environmental factors in different regions of Yilong Lake, Yunnan Province, China. *Huan Jing Ke Xue* **2019**, *40*, 2249-2257 (in Chinese), doi:10.13227/j.hjkx.201810157.

13. Jia, N.; Wang, Y.; Guan, Y.; Chen, Y.; Li, R.; Yu, G. Occurrence of *Raphidiopsis raciborskii* blooms in cool waters: Synergistic effects of nitrogen availability and ecotypes with adaptation to low temperature. *Environ. Pollut.* **2021**, *270*, 116070, doi:10.1016/j.envpol.2020.116070.

14. Wang, X.; Li, Q.; Chen, W.; Han, M.; Han, L.; Brancelj, A. Composition and indication of plankton fatty acids under the influence of environmental factors in the Hongfeng Reservoir, Southwest China. *Aquat. Ecol.* **2022**, 653-666, doi:10.1007/s10452-022-09942-0.

15. Ouyang, T.; Yang, S.; Zhao, L.; Ji, L.; Shi, J.; Wu, Z. Temporal heterogeneity of bacterial communities and their responses to *Raphidiopsis raciborskii* blooms. *Microbiol. Res.* **2022**, *262*, 127098, doi:10.1016/j.micres.2022.127098.

16. Wu, D.; Xu, Z.; Wang, Y.; Gao, Y. Seasonal variation of phytoplankton community structure in Hengshan reservoir. *Shui Sheng Tai Xue Za Zhi* **2012**, *33*, 54-57 (in Chinese), doi:10.15928/j.1674-3075.2012.04.005.

17. Zha, G. Investigation on the ecological factor in *Cylindrospermopsis*

raciborskii bloom in low salty prawn ponds. *Ecol. Sci.* **2009**, *28*, 293-298 (in Chinese), doi:10.3969/j.issn.1008-8873.2009.04.002.

18. Zou, H.; Hu, R.; Han, B. Structure and dynamics of phytoplankton community in Hedi Reservoir, South China. *J. Trop. Subtrop. Botany* **2010**, *18*, 196-202 (in Chinese), doi:10.3969/j.issn.1005-3395.2010.02.014.

19. Lei, M.; Peng, L.; Han, B.; Lei, L. Distribution and affecting factors of *Cylindrospermopsis raciborskii* in Guangdong reservoirs. *Huan Jing Ke Xue* **2018**, *39*, 5523-5531 (in Chinese), doi:10.13227/j.hjkx.201805104.

20. Lei, L.; Peng, L.; Huang, X.; Han, B. Occurrence and dominance of *Cylindrospermopsis raciborskii* and dissolved cylindrospermopsin in urban reservoirs used for drinking water supply, South China. *Environ. Monit. Assess.* **2014**, *186*, 3079-3090, doi:10.1007/s10661-013-3602-8.

21. Zhao, L.; Lei, L.; Peng, L.; Han, B. Seasonal dynamic and driving factors of *Cylindrospermopsis raciborskii* in Zhenhai Reservoir, Guangdong Province. *Hu Po Ke Xue* **2017**, *29,* 193-199 (in Chinese), doi:10.18307/2017.0121.

22. Xue, X.; Fang, G.; Zou, C.; Zhu, D.; Xiao, L.; Peng, L. Diel vertical distribution patterns of *Raphidiopsis raciborskii* in Dashahe Reservoir. *Sheng Tai Xue Za Zhi* **2020**, *39*, 2348-2355 (in Chinese), doi:10.13292/j.1000-4890.202007.032.

23. Lu, Y.; Lei, M.; Ye, J.; Lei, L.; Han, B. Intraspecific variation of morphological traits and toxin-producing capacity and phylogenetic

analysis for *Cylindrospermopsis raciborskii* from Qiandenghu Lake, Guangdong Province. *Hu Po Ke Xue* **2020**, *32*, 144-153 (in Chinese), doi:10.18307/2020.0114.

24. Lei, L.; Lei, M.; Cheng, N.; Chen, Z.; Xiao, L.; Han, B.; Lin, Q. Nutrient regulation of relative dominance of cylindrospermopsin-producing and non-cylindrospermopsin-producing *Raphidiopsis raciborskii*. *Front. Microbiol.* **2021**, *12*, 793544, doi:10.3389/fmicb.2021.793544.
25. Lu, Z.; Lei, L.; Lu, Y.; Peng, L.; Han, B. Phosphorus deficiency stimulates dominance of *Cylindrospermopsis* through facilitating cylindrospermopsin-induced alkaline phosphatase secretion: Integrating field and laboratory-based evidences. *Environ. Pollut.* **2021**, *290*, 117946, doi:10.1016/j.envpol.2021.117946.
26. Tang, H.; Liu, P.; Wu, J.; Liao, H.; He, A. Phytoplankton functional groups and their response to physiochemical factors in Hongchaojiang Reservoir in Guangxi Province. *Shui Sheng Tai Xue Za Zhi* **2022**, 1-13 (in Chinese), doi:10.15928/j.1674-3075.202105120145.
